# The Presence of SFRP1 Reduces the High Risk of Metastasis in RANKL-Expressing Canine Mammary Cancers

**DOI:** 10.3390/ani16060968

**Published:** 2026-03-19

**Authors:** Nina Durys, Joanna S. Morris, Robert Klopfleisch, Torsten Stein

**Affiliations:** 1 Institute of Veterinary Biochemistry, Freie Universität Berlin, Oertzenweg 19b, 14163 Berlin, Germany; n.durys@fu-berlin.de; 2 School of Biodiversity, One Health & Veterinary Medicine, University of Glasgow, 464 Bearsden Rd., Glasgow G61 1QH, UK; joanna.morris@glasgow.ac.uk; 3 Institute of Veterinary Pathology, Freie Universität Berlin, Robert-von-Ostertag-Straße 15, 14163 Berlin, Germany; robert.klopfleisch@fu-berlin.de

**Keywords:** SFRP, RANKL, canine mammary cancer

## Abstract

Canine mammary cancers (CMCs) are one of the most prevalent types of neoplasm in dogs, are frequently malignant, and display high tumour heterogeneity, making evaluating prognosis and predicting successful treatment outcomes difficult. In a previous pilot study, overexpression of the Wnt pathway-associated protein SFRP1 was found to correlate with negative metastasis status in CMCs at both mRNA and protein levels. To establish SFRP1 as a potential biomarker for CMC progression, additional verification of these results in an independent dataset is required, as well as an investigation as to whether SFRP1 expression in CMCs is associated with altered Wnt- or RANKL signalling pathways. In an independent verification cohort of 122 cases of archival CMC FFPE material, expression of SFRP1 was assessed by RT-qPCR and immunohistochemistry. The same tumours were further assessed for RANKL, phosphoROCK2, and NFkB-p65 protein expression. Our data verified that SFRP1 mRNA (*p* = 0.025) was negatively associated with metastasis status; however, differences in protein expression did not reach statistical significance (*p* = 0.139). Neither did SFRP1 significantly correlate with expression of any of the other proteins tested. Instead, a strong association was found for RANKL positivity with increased metastasis status (*p* < 0.001). Co-expression of SFRP1 significantly lowered the higher risk of metastatic spread when compared to RANKL_pos_/SFRP1_neg_ CMCs (*p* = 0.033). Noticeably, all vascular-invasive cell clusters observed in tissue section vessels stained positive for RANKL. Our study identified RANKL expression as a strong marker for cancer progression with a strong link to vascular-invasive cells. However, SFRP1 expression may potentially suppress the pro-metastatic nature of RANKL_pos_ CMCs.

## Simple Summary

Canine mammary cancer is a very common disease affecting about a quarter of unspayed females. Many of these cancers are malignant and metastasise, causing the death of the animal. Accurate estimation of prognosis is often difficult, in part because the criteria used for human breast cancer are not easily applied to the dog. We found that receptor activator of nuclear factor kappa-Β ligand (RANKL), a protein necessary for, among other processes, bone formation and normal mammary gland development, was strongly expressed by metastatic tumours, and could be used as a biomarker for the progression of the disease. In addition, we found that when secreted frizzled-related protein 1 (SFRP1), a protein that can bind to RANKL and inhibit its signalling pathway, was present at the same time, the risk of metastasis was reduced. SFRP1 may therefore play a role in reducing cancer metastasis risk in dogs, at least in part, by suppressing RANKL signalling.

## 1. Introduction

Canine mammary cancers (CMCs) are prevalent and will affect one in four unspayed females. Prevalence varies by region and depends on the frequency of preventive neutering [1]. The only known protective measure is ovariohysterectomy, with the most marked effect seen when it is performed before the first oestrus, and very little effect if performed after 2.5 years of age [2]. Approximately 50% of CMCs are malignant, metastasising most commonly to the lymph nodes and lungs [3].

There are strong morphological similarities between normal human and canine mammary glands and mammary-derived malignancies, with both showing a similar hormone dependency and first presentation at a similar relative age. Metastatic progression via the lymphatic system through local lymph nodes to distant sites is similar in both species; therefore, CMC has been considered a potential model for human breast cancer (HBC). Cancer-associated stroma of CMC and HBC show important similarities, such as upregulation of several known tumour markers like α-Smooth Muscle Actin or Collagen1α1 [4]. Clinical factors such as tumour size and presence of local and distant metastases help predict tumour behaviour [5], with lymph node invasion and metastasis being highly prognostic [6].

Histological grading adapted to the dog is also useful for determining prognosis and is based on tubule formation, nuclear pleomorphism, and the presence of mitotic figures [5]. Other criteria, such as local tissue invasion, absence of myoepithelial proliferation, presence of necrosis and tumour emboli in vessels, may also help determine the degree of malignancy. Despite the similarities between CMC and HBC, important histopathological differences can also be found. In contrast to HBC, CMCs are more heterogeneous, with complex and mixed tumours common in dogs but rare in humans. Further, while HBC is routinely classified according to hormone receptor status, oestrogen receptor (ER) and progesterone receptor (PR) expression in CMCs has been contradictory [6], making prognostication more challenging [5]. A high-risk group gene signature has been described for canine mammary tumours [7].

Classification of CMCs as ER-, PR-, and human epidermal growth factor receptor 2 (HER2)-positive or -negative is not routinely performed, partly due to cost, but also due to lack of consensus on what hormone receptor status best relates to prognosis for the canine disease [8]. Canine mammary tumours can be classified, similarly to HBC, as luminal A or basal-like, based on their molecular subtype; however, classification into luminal B and HER2-enriched subtypes is unreliable [9]. There is also a lack of standardised antibodies for canine tissue that could be used similarly to human diagnostic pathology. Hence, no reliable prognostic biomarkers for the canine disease currently exist.

In a previous pilot study, Secreted Frizzled-Related Protein 1 (SFRP1) was found to be negatively associated with metastasis status in CMCs [10], similar to HBC [11,12], where Sfrp1 is epigenetically downregulated and has been proposed as a tumour suppressor [13]. However, because of the relatively small size of the previous cohort, a verification of these results in a larger independent dataset was necessary to confirm SFRP1 as a biomarker for metastasis formation in CMC. Furthermore, the mechanism by which SFRP1 acts within the tumour microenvironment in these cancers in vivo is still not known.

SFRP1 plays a role in several cancer-associated pathways, notably the Wnt and Receptor activator of NFκB/ligand (RANK/RANKL) pathways [14,15]. All five members of the SFRP family possess a conserved cysteine-rich domain (CRD), which is structurally similar to the CRD of Frizzled (Fzd) receptors. They can therefore block the Wnt pathway by competing with FZD for the binding of signalling proteins [16].

SFRP1 is an inhibitor of proliferation that inhibits Wnt signalling in several cell types, including epidermal progenitor cells [17]. SFRP1 is repressed by c-Myc, and Myc-induced mammary tumours showed both downregulated SFRP1 and upregulated expression of Wnt target genes in what has been proposed as a positive feedback loop between c-Myc and Wnt pathways in breast cancer [18]. The Wnt/β-catenin pathway is dysregulated in human, canine and feline mammary gland tumours, with some tumours showing decreased levels of membrane β-catenin staining [10,19]. However, no significant correlation was previously found between the level of SFRP1 expression and β-catenin intracellular localisation or activation of the canonical Wnt pathway by immunohistochemistry (IHC) [10], even though reduced β-catenin membrane staining was strongly associated with metastasis formation. It is therefore unclear how the reduction in SFRP1 translates into altered signalling pathways in these cells. In order to understand how SFRP1 acts as a tumour suppressor in CMC and which signalling pathways may be perturbed in cancers with reduced SFRP1 levels, there was a need to further describe these CMCs in relation to the activity of pathways that are known to be affected by SFRP1.

In addition to the β-catenin-dependent canonical Wnt pathway, Wnt binding to the FZD receptor can also activate the non-canonical (PCP) Wnt pathway, leading to phosphorylation of ROCK2 and enhanced remodelling of the actin cytoskeleton [16]. Similarly, activation of the RANK/RANKL pathway causes accumulation of RANKL in the cell membrane, inducing shuttling of the transcription factor NfκB from the cytoplasm to the nucleus. Both pathways have previously been shown to be suppressed by SFRP1 [15,16].

In this study, we first aimed to verify our earlier results in a larger independent data set of SFRP1 downregulation in metastatic CMCs at both mRNA and protein levels. Using immunohistochemistry, we further investigated whether other potential SFRP1-associated downstream pathways were affected by the presence or absence of SFRP1 in these CMCs. For this, we stained the same CMCs for RANKL, phosphoROCK2 (pROCK2), and NFκB as factors potentially affected by SFRP1 to investigate a potential link to metastasis formation.

## 2. Materials and Methods

### 2.1. Tissues

Formalin-fixed paraffin-embedded (FFPE) canine mammary gland primary cancer tissue was obtained from the archive of the Institute of Veterinary Pathology. The tissues were collected for diagnostic purposes in the years 2016–2024 and reviewed for cancer grade according to the Peña histological grading system [5] and metastases to lymph nodes or vessels by two veterinary pathologists. For all samples chosen for this study, clinical information on lymph node status was available, and this was the criterion used to group the samples by metastasis status. Cases that presented with lymph nodes and vessels free of tumour cells were classified as non-metastatic, while cases that presented with tumour cell invasion in either lymph nodes or lymph vessels were classified as metastatic. Cases for which such information was missing were not used for analysis. Information on distant metastases (e.g., lungs, bones or other organs) was not available for most cases. The same cases from each dog were used for RNA isolation and IHC, where possible. An additional set of 62 cases was used for IHC only.

### 2.2. RNA Isolation

Five 10 μm sections were cut from each FFPE block using a microtome. The microtome and blades were pre-treated with RNAse Away (Thermo Fisher Scientific, Kalamazoo, MI, USA) before cutting each block. RNA was isolated using a NucleoSpin Total RNA FFPE Kit (Macherey & Nagel, Düren, Germany) according to the manufacturer’s protocol, including an on-column DNAse treatment step. RNA was eluted in 15 μL nuclease-free water and stored at −20 °C for short-term storage (for use within 24 h) or −80 °C for long-term storage. Nucleic acid concentration and purity were assessed on a NanoDrop device (ND-1000, PEQLAB Biotechnologie GmbH, Erlangen, Germany) and on a Bioanalyzer using the RNA 6000 Pico Kit (Agilent, Santa Clara, CA, USA).

### 2.3. RT-qPCR

cDNA was synthesised from 1 μg of each RNA sample using a LunaScript RT SuperMix Kit (NEB, Ipswich, MA, USA) according to the manufacturer’s protocol. A negative control without reverse transcriptase was prepared for each sample. cDNA was stored at −20 °C.

Primers and fluorescent probes for *Sfrp1* and *Rps19* (housekeeping control) were designed to be exon-spanning to avoid amplifying genomic DNA and have been described previously [10]. Briefly, probes were dual-labelled with 6-FAM and BHQ1 (Merck, Darmstadt, Germany). qPCR was performed in triplicate with one no-RT control for each sample in 10 μL reactions using a LunaScript Universal Probe qPCR Mix (NEB), according to the manufacturer’s protocol. Primers and probes were used at a concentration of 0.4 μM and 0.2 µM, respectively. The PCR reaction proceeded as follows: 95 °C for 1 min, followed by 45 cycles of 95 °C for 15 s, 60 °C for 30 s. Abundance of *Sfrp1* mRNA was calculated relative to the expression of *Rps19* using the formula $\frac{1}{2^{∆Ct}}\times 100\%$, which describes the total level of abundance in addition to the relative fold-change between samples. Results were accepted for all samples that amplified before Ct = 35. If the Ct value for *Rps19* was 35 or higher, the sample was not used for analysis.

### 2.4. Immunohistochemistry

FFPE blocks were cut into 2–4 μm sections by microtome, collected in a cold-water bath (20 °C), stretched in a hot-water bath (45 °C), and then placed on adhesion-silanised microscope slides. The sections were dried overnight and then dewaxed in xylene 2× for 10 min, followed by 100% ethanol 2× for 2 min, 96% ethanol 2× for 2 min, and 70% ethanol 1× for 2 min. The antigen retrieval method and antibody dilution for each of the antibodies used are presented in Table 1. The slides were processed manually using a Shandon Sequenza Coverslip system (Thermo Fisher Scientific) for handling. The primary antibody was incubated at 4 °C overnight. A Super Sensitive™ negative control antibody (Biogenex, Fremont, CA, USA), appropriate to the species from which the primary antibody was derived, was used as the control. One negative control slide was used for each instance of IHC performed (per analyst, per day, per antibody).

Endogenous peroxidase blocking was performed before the application of the primary antibody for samples processed in citrate buffer or afterwards for those processed in Tris/EDTA buffer.

Following three washes, a species-appropriate secondary antibody diluted 1:200 in PBS with normal goat serum was applied and incubated for 30 min at RT. Next, an avidin-biotin–peroxidase complex (ABC) was prepared according to the kit manufacturer’s instructions (Vectastain ABC-HRP Kit, Vector Laboratories, Newark, CA, USA) and applied for 30 min. The samples were stained using a DAB solution for 8 min with shaking and the nuclei counterstained with haematoxylin for 1 min, except for the NFκB p65 samples, which were counterstained for 5 s only. The sections were dehydrated in a series of alcohol baths with ascending concentration and finally in xylene, followed by mounting and scanning using an Aperio ScanScope Slide Scanner (Leica Biosystems, Wetzlar, Germany) at 200× or 400× magnification. The samples were then classified into no, weak, moderate or strong signal (a score of 0, 1, 2, or 3, respectively) by evaluating the proportion of tumour cells exhibiting the signal and the signal intensity using the QuickScore method [20]. In short, the percentage of tumour area exhibiting signal positivity was ranked from 1 to 6, with a score of 1 signifying 0–5% of positive area, 2—5–19%, 3—20–39%, 4—40–59%, 5—60–79%, and 6—80–100%. Staining intensity was evaluated on a scale from 0 (none) to 3 (strong), and the final score was obtained by multiplying these two values. Then, samples were assigned to the 0–3-point scale, where a multiplicative value of 0 gave a score of 0, 1–6 of 1, 8–12 of 2, and 15–18 of 3. In the case of SFRP1, myoepithelial and stromal signals were considered for the evaluation. For RANKL and pROCK2, only cytoplasmic signal was considered. For NFκB, only the nuclear signal was considered.

### 2.5. Data Analysis and Statistics

Statistical analyses were performed using SPSS software (IBM SPSS Statistics, version 29.0.0.0). A Mann–Whitney U test was performed to evaluate distributions of values grouped by their metastasis status. For estimating correlations, the Pearson correlation coefficient was calculated with a confidence interval of 95%. Additionally, *p*-values of <0.05 were considered statistically significant.

## 3. Results

### 3.1. Expression of Sfrp1 mRNA Correlates Negatively with Metastasis Status

**Figure 1:**
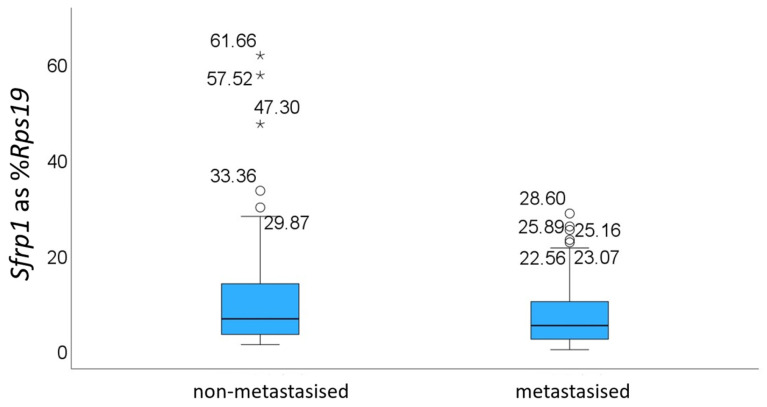
*Sfrp1* RNA abundance in CMCs displayed as a % of the expression of the housekeeping gene *Rps19* and grouped by metastasis status. The box and whiskers represent the quartile distribution, where the central bar represents the median and the individual outlier points (°, *) are labelled with their value. Outlier points labelled with asterisks (*) denote extreme values (3 interquartile ranges above the 3rd quartile). Mann–Whitney U test: *p* = 0.078.

A set of 58 non-metastasised and 63 metastasised samples, independent of our previous study, were initially identified from the tissue archive of the Institute for Veterinary Pathology and chosen for RNA extraction. Samples were excluded from analysis if the RNA isolation process yielded RNA of too low concentration (<20 ng/µL) or if the resulting cDNA failed to amplify for *Rps19*. A total of 35 non-metastasised and 52 metastasised FFPE blocks were therefore finally used for analysis (Figure 1). As previously observed, *Sfrp1* expression was higher in non-metastasised tumours (median value of 6.56% for non-metastasised vs. 5.16% for metastasised), but this was not statistically significant (*p* = 0.078). Mean values differed similarly (mean 13.01% +/− SD 15.54 vs. 7.61% +/− SD 7.54), but were skewed by the presence of high outliers, including three samples with very high values in the non-metastasised samples. As previously done (10), *Sfrp1* mRNA expression was grouped into four groups—0 (samples expressing <1% of *Sfrp1* as % of *Rps19*), 1 (1–10%), 2 (10–25%), and 3 (>25%)—corresponding to no, low, medium and high expression. When grouped in this way and consistent with our previous data, *Sfrp1* expression was associated with metastasis, with a *p*-value of 0.025.

**Figure 2:**
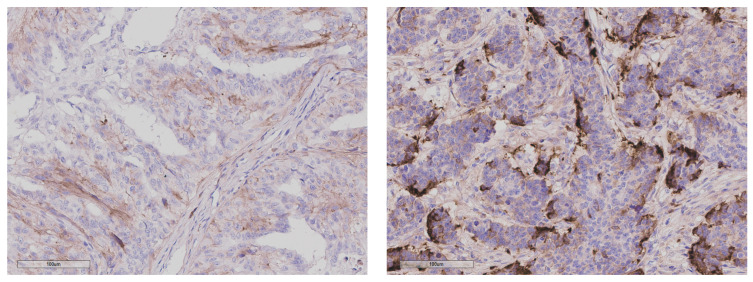
Examples of the observed stromal staining pattern for SFRP1 in two tumour samples with low (score 1, **left**) and high (score 3, **right**) expression.

**Figure 3:**
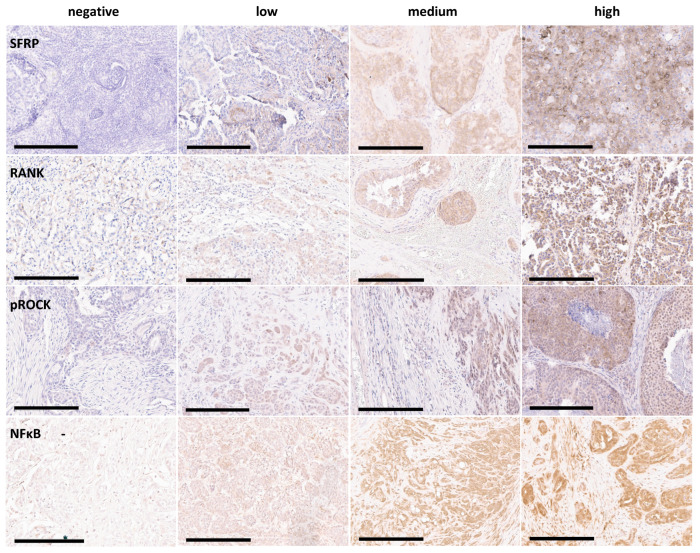
IHC staining for SFRP1, RANKL, pROCK2, and NFκB-p65. Examples of negative, low, medium and high abundance are given for each protein analysed. 200× magnification. The black bars represent 200 μm.

To verify the association of SFRP1 expression with metastasis status at the protein level, 64 and 68 FFPE blocks of non-metastasised and metastasised CMCs, respectively, were initially identified and chosen for IHC staining. Samples were excluded from analysis if they detached during the staining process. No samples had to be excluded because of negative control failure. Ultimately, 115 samples (60 metastasised and 55 non-metastasised) were included in the analysis for SFRP1 protein expression. For SFRP1, both myoepithelial and stromal staining were considered for the evaluation, as shown in Figure 2. The abundance of SFRP1 protein was classified as negative, low, medium or high (a score of 0, 1, 2, or 3, respectively; Figure 3).

IHC results and statistical analyses are summarised in Table 2. Surprisingly, SFRP1 protein expression was not statistically significant, with a Mann–Whitney U *p*-value of 0.139 and a Pearson correlation *p* = 0.129 (correlation −0.143, 95% CI [−0.317–0.042]), which includes 0.

### 3.2. Expression of RANKL Protein Correlates Positively with Metastasis Status

To test any association of the pathway proteins RANKL, pROCK2, and NFκB-p65 with metastasis and SFRP1 status, FFPE blocks of non-metastasised and metastasised primary CMCs were chosen, as described above (the same 64 and 68 FFPE blocks of non-metastasised and metastasised CMCs, respectively, were stained for each protein). A total of 33 samples (16 non-metastasised, 17 metastasised) analysed for SFRP1 expression in a previous study [6] were added to this sample cohort in order to increase the statistical power, with a final set consisting of 74 non-metastasised and 80 metastasised samples chosen for analysis, each sample for at least one of the three proteins.

The abundance of each protein was classified as negative, low, medium or high (a score of 0, 1, 2, or 3, respectively; Figure 3). For RANKL and pROCK2, only the cytoplasmic signal was considered, and for NFκB-p65, only the nuclear signal.

No significant relationship was found between the expression levels of SFRP1, pROCK2 and NFκB-p65, RANKL and pROCK2 or NFκB-p65, nor did the expression of pROCK2 or NFκB-p65 significantly correlate with metastasis status. In contrast, RANKL expression was strongly associated with metastasis status (Mann–Whitney U: *p* < 0.001) and also with a current best-practice progression marker, histological tumour grade (Pearson coefficient 0.282, *p* = 0.002) (Supplementary Table S1). The results of the statistical analyses for metastasis status are shown in Table 3.

To further test this association, samples were grouped into either RANKL-negative vs. positive (IHC values of 0 vs. 1, 2 or 3) or RANKL low- vs. high-expressing groups (IHC values 0, 1 vs. 2, 3). RANKL positivity (RANKL_pos_) was positively correlated with metastasis status (Pearson correlation 0.195, *p* = 0.023), and comparing negative/low to medium- or highly expressing tumours showed a higher positive correlation (0.384, *p* < 0.001) with metastasis status.

### 3.3. Expression of SFRP1 Is Negatively Correlated with Metastasis Status in RANKL_pos_ Tumours

Though SFRP1 expression was associated with negative metastasis status in CMCs, the suppressive mechanism for this has not yet been established. In contrast to SFRP1, RANKL expression in our cohort of CMCs was very strongly associated with positive metastasis status. If a suppressive effect by SFRP1 on metastasis formation involved interference with RANKL signalling, then it could be reasonably expected that RANKL-positive tumours with higher levels of SFRP1 may have a lower risk of metastatic spread than those negative for SFRP1. To test this, the RANKL_pos_ subgroup (IHC value > 0) was analysed separately. This included a total of 121 CMCs (metastasised: n = 68, non-metastasised: n = 53).

RANKL_pos_ samples were then isolated from the cohort, and the association of SFRP1 expression level with metastasis status was analysed using a Mann–Whitney U test as well as by Pearson correlation (Table 4).

Indeed, this cohort could be significantly stratified by *Sfrp1* mRNA expression (Mann–Whitney U test, *p* = 0.013) as well as SFRP1 protein expression (Mann–Whitney U test, *p* = 0.033) with a Pearson correlation coefficient of −0.201 (*p* = 0.026). Therefore, it is possible that SFRP1 may at least in part assert its protective role via suppressing the pro-metastatic effect of RANKL.

### 3.4. Expression of RANKL in Vascular-Invasive Cells Is High, Irrespective of the Expression Pattern in the Surrounding Tumour

Circulating tumour cells (CTCs) are tumour cells that have detached from the tumour mass and entered circulation, either in the blood or lymph vessels. They are the primary means for the tumour to metastasise, and are characterised by a higher rate of EMT than the main tumour mass [21]. CTCs are generally rare in cancer patients, and techniques for their analysis usually require enrichment, but if present in large quantities, they may be observed during histopathology observation either as single cells or cell clumps. Without further analysis, it is unknown whether vascular-invasive cells observed in a histopathological slide are in fact circulating tumour cells or have only infiltrated the vasculature locally. However, the presence of such cells is indicative of higher tumour malignancy.

**Figure 4:**
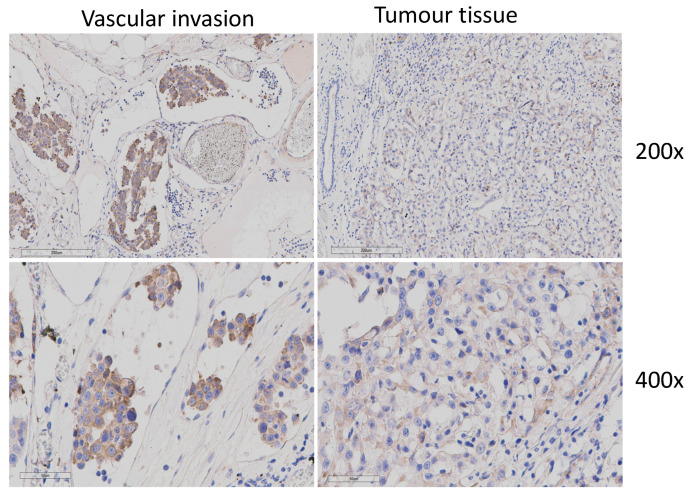
RANKL staining in vascular invasion and corresponding tumour at 200× (**top**) and 400× (**bottom**) magnification in the same tissue section.

Out of the 80 metastatic tumours analysed, 22 showed vascular-invasive cells within local blood or lymph vessels, indicative of possible metastasis. Blood and lymph vessels were identified by morphology and the presence or absence of erythrocytes, respectively. When these tumours were stained for RANKL, all vascular-invasive cells were found to express high levels of RANKL (score 2 and 3). Noticeably, there was no direct correlation between the level of RANKL expression of the tumour and that of the cells found in the neighbouring tissue (Pearson coefficient 0.108, *p* = 0.633), as the vascular-invasive cells from CMCs with low RANKL expression were still strongly positive (Figure 4).

**Figure 5:**
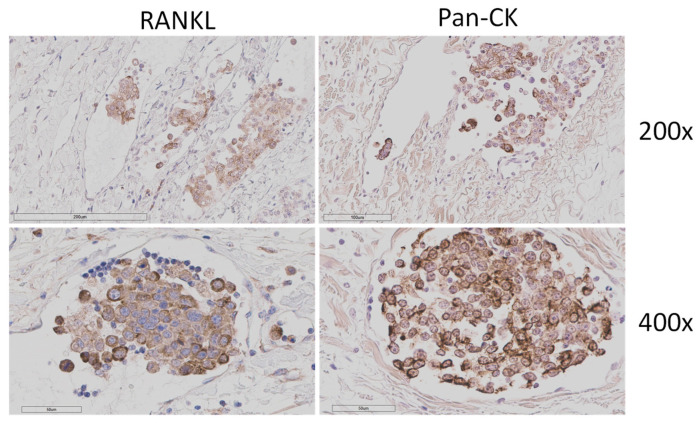
RANKL and pan-cytokeratin (CK) staining of consecutive sections from the same representative vascular-invasive cell sample at 200× (**top**) and 400× (**bottom**) magnification.

To verify whether these cells in vessels were in fact tumour cells, we performed pan-cytokeratin staining on 18 samples: 7 metastatic CMCs without such cells and 10 with observed vascular-invasive cells. One sample considered to contain vascular-invasive cells was subsequently re-evaluated as negative, as pan-cytokeratin staining showed that the cells were inside a duct, not a lymph or blood vessel. None of the seven samples without vascular-invasive cells showed pan-cytokeratin-positive cells within vasculature, as expected. Out of the nine samples considered to contain vascular-invasive cells, all showed pan-cytokeratin positivity (Figure 5).

## 4. Discussion

Our study aimed to validate our earlier finding that SFRP1 is associated with metastasis status in CMCs by using a larger sample size with better predictive power, to assess downstream pathway activation by SFRP1 using IHC, and therefore to determine whether SFRP1 could be a viable biomarker for metastasis progression in CMCs. An association between SFRP1 expression, at both the mRNA and protein levels, and negative metastasis status could be verified. However, the association was weak, making SFRP1 unsuitable as a general prognostic marker. One study found a significant upregulation of *Sfrp1* mRNA in non-metastasised canine mammary tumours; however, it did not find downregulation of *Sfrp1* in metastasised tumours when compared to normal stroma [22]. It is possible that the differences in SFRP1 expression patterns, such as intensity of staining and epithelial or stromal localisation, are related to tumour origin or hormone receptor status, and that these factors are more responsible for metastasis formation than SFRP1 expression itself. The typical staining pattern for SFRP1 that we observed in most SFRP1-positive IHC samples shows SFRP1 both within the cytoplasm of tumour cells and secreted into the stroma.

So far, SFRP1’s role as a tumour suppressor in cancer has mostly been ascribed to its interaction with WNT proteins and their receptors [23]. We have previously not been able to show an association between reduced canonical Wnt signalling and SFRP1 abundance. Though our previous data did show an association between reduced β-catenin membrane staining and metastasis status, we did not see the preferential nuclear staining, as has been reported previously in human breast cancer [24]. However, others, in agreement with our data in canine mammary tumours, also reported reduced membrane staining without enhanced nuclear staining in some breast cancers [25]. The Wnt pathway was dysregulated in canine mammary cancer stroma when compared to normal tissue in a transcriptomic study, with upregulation of *Sfrp1* noted [12]. In another study, *Sfrp2* was upregulated in both malignant and benign canine mammary tumours, and connected to Wnt pathway dysregulation [26]. It is possible that the effect of SFRP1 on metastasis is restricted to a subgroup of all CMCs. Our current study was also not able to detect a relationship between SFRP1 and NFκB staining, even though NFκB is a RANK target and downstream of RANKL in the signalling pathway, nor pROCK2 staining, as might be expected if SFRP1 suppressed the non-canonical Wnt pathway activity in CMCs, as it has been suggested for human breast cancer [27,28]. In fact, only one sample showed a complete localised inverse relationship between SFRP1 and pROCK2, with SFRP1 positive areas being negative for pROCK2 and vice versa. Instead, SFRP1 expression was able to significantly stratify RANKL-positive tumours by metastasis status. Direct interaction between these two proteins has been shown for SFRP1’s inhibition of osteoclast formation [15]. SFRP1^−/−^ mice show significantly elevated RANKL expression in the mammary gland, leading to premature development [29]; however, little is known about the effects of SFRP1 on RANKL in cancer. Our results strongly indicate that SFRP1’s tumour suppressor activity might, at least in part, act through interference with RANKL signalling.

RANKL is expressed in osteoblasts that regulate skeletal development [30] and is a mediator of progesterone signalling during normal mammary ductal side-branching [31], in normal human breast tissue [32], and in early breast tumorigenesis in a MMTV-*neu* mouse tumour model [33]. It is also implicated in bone metastasis formation [34] through RANKL-expressing osteoblasts displaying chemoattraction towards RANK-positive circulating breast cancer cells, as well as through RANKL-positive CTCs having a proliferation and survival advantage [35]. The RANK/RANKL pathway has been found to be upregulated in high-grade canine mast cell tumours [36], while soluble RANKL has been indicated to promote bone metastasis in mice [37]. Moreover, epithelial RANK, though not RANKL [38], is strongly expressed by a high percentage of carcinomas [39] and is a poor prognostic factor in human breast cancer [40,41]. It is not possible to draw conclusions on whether there is no direct effect of SFRP1 on RANKL in the context of CMC, or if it remains unobservable using IHC techniques. As RANKL causes an increase in proliferation of the mouse mammary epithelium when exposed to progesterone [33], knowledge about PR status in CMCs could yield further insights. However, IHC for PR in dogs yielded unsatisfactory results due to a lack of canine-specific antibody.

In our dataset, RANKL was very strongly associated with metastasis and tumour grade, and all vascular-invasive cells stained positive for RANKL, making it a good candidate as a prognostic marker for risk of CMC metastasis formation. Whether RANKL is a stronger predictor than grade or the presence of vascular-invasive cells locally remains unclear, as all three are strongly associated with metastasis. However, RANKL expression and grade appear to be independent, as these groups do not significantly overlap (Cramer’s V 0.213, *p* = 0.104). Therefore, incorporating RANKL expression in diagnostics may add value to predicting metastasis formation. Within our cohort of CMCs, most exhibited only moderate or minimal tubule formation, by which they received a higher malignancy ranking using the Pena histological grading system [5]. Conversely, even aggressive tumours with high pleomorphism frequently exhibited fewer than 10 mitoses per 10 high-power fields, which would score lower in the grading system. Grading canine mammary tumours using the accepted grading system and only these three criteria can lead to uncertainty when evaluating the likelihood of metastasis and disease progression; therefore, an additional marker such as RANKL may be useful.

RANKL is expressed either as a transmembrane protein or cleaved to a soluble form [42]. The soluble form may then interact with the receptor. RANKL is expected to localise to the membrane in its bound-to-receptor form. This was not observed in our study. Rather, a diffuse cytoplasmic staining combined with moderate membrane staining was the most common pattern. Therefore, it is difficult to judge whether this staining pattern indicates whether the RANKL pathway is more or less active in metastasised vs. non-metastasised CMCs. A similar staining pattern was demonstrated in a study on young breast cancer patients [43], where higher RANKL expression was found to be correlated with pregnancy. RANKL was detected in neoplastic epithelium as well as in the stroma in a mouse model of mammary gland tumours [33].

However, it was striking that RANKL expression was found at medium or high levels in all vascular-invasive cells and clusters in all samples with observable local vascular invasion on H&E, irrespective of the RANKL staining intensity of the tumour itself. Whether this RANKL expression is a cause for or a response to vascular invasion is unclear. Nevertheless, its expression might be advantageous to tumour cell survival within vessels.

The RANKL-RANK-OPG system is essential for the correct functioning of the immune system. RANKL is normally expressed on T cells, with its downregulation causing an autoimmune disease through a lack of tolerance of lymphoid tissues to T cells [44]. RANKL expression could therefore possibly help tumour cells evade immune response. Denosumab, an anti-RANKL antibody, has been used in the treatment of human breast cancer with bone metastases, and the expression of RANK by CTCs in HBC determines the effectiveness of denosumab treatment [34]. Although RANK expression is a negative prognostic factor for breast cancer patients [45], its expression is required for denosumab’s effectiveness. If RANKL contributes to metastasis formation in dogs by giving CTCs a survival advantage, denosumab (or a canine equivalent) might also be suitable for the cancer treatment of dogs. Denosumab recognises the T233 to Y241 sequence on RANKL [46], which is 100% conserved between dog and human. However, as it is a fully human antibody, unintended immune reactions may occur in dogs upon administration.

Interestingly, one case of canine mammary sarcoma (not included in our data) displayed strong RANKL positivity in vascular-invasive cells, but not in the tumour mass itself. In dogs, mixed mammary tumours composed of epithelial cells and cartilage or bone tissue are common and well described [47]. The estimated prevalence of canine mammary sarcomas is in the range of 3.5–8.3%, with osteosarcomas even rarer at 1% prevalence [48]. In contrast, sarcomas of the breast are rare in humans [49], where they often display epithelial markers and metastasise through the lymph nodes, and are hence highly likely to originate from the epithelial component [50]. Canine sarcomas have a poor prognosis, and 75% of them are metastatic [48]. Differentiation of malignant mixed tumours in the dog, which are composed of carcinomatous and sarcomatous regions, from complex carcinomas, is difficult [51]. RANKL expression by vascular-invasive cells may not be specific only to carcinomas, but may occur regardless of tumour type.

The bone is one of the primary sites for breast cancer metastasis [35]. Breast cancer metastasises to the bone in HBC more often than in CMC (at a rate of 60–70% vs. 5–11%), but among dogs with bone metastases, the mammary gland is the most common primary tumour site [52]. Case studies find higher incidence rates of bone metastasis in primary CMCs of about 20% [52,53], and it has been argued that the incidence rate of bone metastases in dogs may be underrepresented due to the rarity of skeleton screening in canine patients [52]. The relationship between CMC, RANKL expression and skeletal dysplasia would be an interesting direction of further study; however, for the canine patients who made up our study cohort, information on bone events is rarely recorded.

Therefore, the relationship between tumour origin, cellular subtype, and the mechanism of metastasis formation in dogs bears further investigation.

## 5. Conclusions

Our study identified RANKL expression as a marker of cancer progression with a strong link to vascular-invasive cells. Consistent with previous results, our data verified SFRP1_neg_ status as correlated with metastasis formation in all CMCs, especially in conjunction with RANKL status. RANKL_pos_/SFRP1_pos_ CMCs had a significantly lower risk of metastatic spread when compared to RANKL_pos_/SFRP1_neg_ CMCs. SFRP1 expression may potentially suppress the pro-metastatic nature of RANKL_pos_ CMCs.

## Figures and Tables

**Table 1:** Antibodies used for IHC with antigen retrieval method and dilution.

Antibody	Antigen Retrieval	Antibody Dilution	Manufacturer	Species Host
SFRP1	Tris/EDTA pH 9, 15 min 600 W in microwave	1:200	Abcam (Cambridge, UK), 126613	Monoclonal Rabbit
RANKL	Citrate buffer, 12 min 600 W in microwave	1:200	Affinity Biosciences (Cincinnati, OH, USA), AF0313	Polyclonal Rabbit
pROCK2	Citrate buffer, 12 min 600 W in microwave	1:100	Invitrogen (Darmstadt, Germany), PA 5-34895	Polyclonal Rabbit
NFκB p65 (NFκB)	Citrate buffer, 12 min 600 W in microwave	1:100	Invitrogen (Darmstadt, Germany), PA 5-16545	Polyclonal Rabbit

**Table 2:** Summary of qPCR and IHC results displaying the expression levels of SFRP1, grouped by metastasis status. The *p*-values were calculated using a Mann–Whitney U nonparametric two-independent-sample test. Correlation was calculated using a bivariate two-tailed Pearson test.

	Metastasis Status (*n*, %)			Mann– Whitney U	Pearson Correlation		
	Negative	Positive	Total	*p*-Value	Coefficient	95% CI	*p*-Value
*Sfrp1* mRNA as % of *Rps19*	35 (40.2%)	52 (59.8%)	87 (100%)	0.078	−0.215	−0.402–−0.010	0.040
*Sfrp1* mRNA as % of *Rps19* grouped							
0	0 (0%)	7 (12.7%)	7 (7.6%)				
1	21 (56.8%)	33 (60%)	54 (58.7%)				
2	10 (27%)	11 (20%)	21 (22.8%)				
3	6 (16.2%)	4 (7.3%)	10 (10.9%)				
TOTAL	37 (100%)	55 (100%)	92 (100%)	0.025	−0.238	−0.422–−0.035	0.022
SFRP1							
0	6 (10.9%)	10 (16.7%)	16 (13.9%)				
1	21 (38.2%)	26 (43.3%)	47 (40.9%)				
2	15 (27.3%)	16 (26.7%)	31 (27%)				
3	13 (23.6%)	8 (13.3%)	21 (18.3%)				
TOTAL	55 (100%)	60 (100%)	115 (100%)	0.139	−0.143	−0.317–0.042	0.129

**Table 3:** Summary of IHC results displaying the expression levels of RANKL, pROCK2, and NFκB grouped by metastasis status. The *p*-values were calculated using a Mann–Whitney U nonparametric two-independent-sample test. Correlation was calculated using a bivariate two-tailed Pearson test.

	Metastasis Status (*n*, %)			Mann–Whitney U	Pearson Correlation		
	Negative	Positive	Total	*p*-Value	Coefficient	95% C.I.	*p*-Value
RANKL							
0	7 (10.6%)	1 (1.4%)	8 (5.9%)				
1	26 (39.4%)	9 (12.9%)	35 (25.7%)				
2	31 (47%)	30 (42.9%)	61 (44.9%)				
3	2 (3%)	30 (42.9%)	32 (23.5%)				
TOTAL	66 (100%)	70 (100%)	136 (100%)	<0.001	0.503	0.366–0.619	<0.001
pROCK2							
0	40 (66.7%)	37 (55.2%)	77 (60.6%)				
1	15 (25%)	24 (35.8%)	39 (30.7%)				
2	4 (6.7%)	5 (7.5%)	9 (7.1%)				
3	1 (1.7%)	1 (1.5%)	2 (1.6%)				
TOTAL	60 (100%)	67 (100%)	127 (100%)	0.231	0.085	−0.090–0.256	0.341
NFkB p65							
0	14 (23%)	13 (19.4%)	27 (21.1%)				
1	21 (34.4%)	28 (41.8%)	49 (38.3%)				
2	20 (32.8%)	22 (32.8%)	42 (32.8%)				
3	6 (9.8%)	4 (6%)	10 (7.8%)				
TOTAL	61 (100%)	67 (100%)	128 (100%)	0.823	−0.023	−0.196–0.151	0.793
RANKL-positive (1–3)	59 (46.1%)	69 (53.9%)	128 (100%)	0.024	0.195	0.028–0.352	0.023
RANKL strongly positive (2–3)	33 (35.5%)	60 (64.5%)	93 (100%)	<0.001	0.384	0.230–0.519	<0.001

**Table 4:** Summary of SFRP1 statistical analysis results for CMCs with positive RANKL expression. The *p*-values were calculated using a Mann–Whitney U nonparametric two-independent-sample test. Correlation was calculated using a bivariate two-tailed Pearson test.

Correlation with Metastasis Status	Mann–Whitney U	Pearson Correlation		
	*p*-Value	Coefficient	95% CI	*p*-Value
*Sfrp1* mRNA as % of *Rps19*	0.013	−0.238	−0.411–−0.048	0.015
*Sfrp1* mRNA as % of *Rps19* grouped	0.007	−0.260	−0.430–−0.072	0.007
SFRP1 protein	0.033	−0.201	−0.366–−0.025	0.026

## Data Availability

The raw data supporting the conclusions of this article will be made available by the authors upon request.

## Supplementary Materials

The following supporting information can be downloaded at: https://www.mdpi.com/article/10.3390/ani16060968/s1, Table S1: Full dataset.

## Abbreviations

The following abbreviations are used in this manuscript:

ABC	Avidin-Biotin Complex
CI	Confidence Interval
CMC	Canine Mammary Cancer
CRD	Cysteine-Rich Domain
CTC	Circulating Tumour Cell
ER	Oestrogen Receptor
FFPE	Formalin-Fixed, Paraffin-Embedded
HBC	Human Breast Cancer
HER2	Human Epidermal growth factor Receptor 2
IHC	Immunohistochemistry
NFκB	Nuclear Factor Kappa-Light-Chain-Enhancer of Activated B cells
OPG	Osteoprotegerin
PCP	Planar Cell Polarity
PR	Progesterone Receptor
qPCR	Quantitative Polymerase Chain Reaction
RANKL	Receptor Activator of Nuclear Factor Kappa-Β Ligand
ROCK2	Rho Associated Coiled-Coil Containing Protein Kinase 2
SFRP1	Secreted Frizzled-Related Protein 1
